# Assessment of changes in autophagic vesicles in human immune cell lines exposed to nano particles

**DOI:** 10.1186/s13578-021-00648-8

**Published:** 2021-07-16

**Authors:** Christopher A. W. David, M. Estela del Castillo Busto, Susana Cuello-Nuñez, Heidi Goenaga-Infante, Michael Barrow, David G. Fernig, Patricia Murray, Matthew J. Rosseinsky, Andrew Owen, Neill J. Liptrott

**Affiliations:** 1 Immunocompatibility Group, Department of Molecular and Clinical Pharmacology, Institute of Translational Medicine, University of Liverpool, Liverpool, UK; 2 Centre of Excellence in Long-Acting Therapeutics (CELT), University of Liverpool, Liverpool, UK; 3 National Measurement Institute, LGC Limited, Queens Road, Teddington, Middlesex, TW11 0LY UK; 4 Department of Chemistry, University of Liverpool, Liverpool, UK; 5 Department of Biochemistry, Institute of Integrative Biology, University of Liverpool, Liverpool, UK; 6 Department of Cellular and Molecular Physiology, University of Liverpool, Liverpool, UK; 7 Centre for Preclinical Imaging, University of Liverpool, Liverpool, UK

**Keywords:** Autophagy, Nanomaterials, Nanotoxicology

## Abstract

**Background:**

Safe and rational development of nanomaterials for clinical translation requires the assessment of potential biocompatibility. Autophagy, a critical homeostatic pathway intrinsically linked to cellular health and inflammation, has been shown to be affected by nanomaterials. It is, therefore, important to be able to assess possible interactions of nanomaterials with autophagic processes.

**Results:**

CEM (T cell), Raji (B lymphocyte), and THP-1 (human monocyte) cell lines were subject to treatment with rapamycin and chloroquine, known to affect the autophagic process, in order to evaluate cell line-specific responses. Flow cytometric quantification of a fluorescent autophagic vacuole stain showed that maximum observable effects (105%, 446%, and 149% of negative controls) were achieved at different exposure durations (8, 6, and 24 h for CEM, Raji, and THP-1, respectively). THP-1 was subsequently utilised as a model to assess the autophagic impact of a small library of nanomaterials. Association was observed between hydrodynamic size and autophagic impact (r^2^ = 0.11, p = 0.004). An ELISA for p62 confirmed the greatest impact by 10 nm silver nanoparticles, abolishing p62, with 50 nm silica and 180 nm polystyrene also lowering p62 to a significant degree (50%, 74%, and 55%, respectively, p < 0.05).

**Conclusions:**

This data further supports the potential for a variety of nanomaterials to interfere with autophagic processes which, in turn, may result in altered cellular function and viability. The association of particle size with impact on autophagy now warrants further investigation.

**Graphic abstract:**

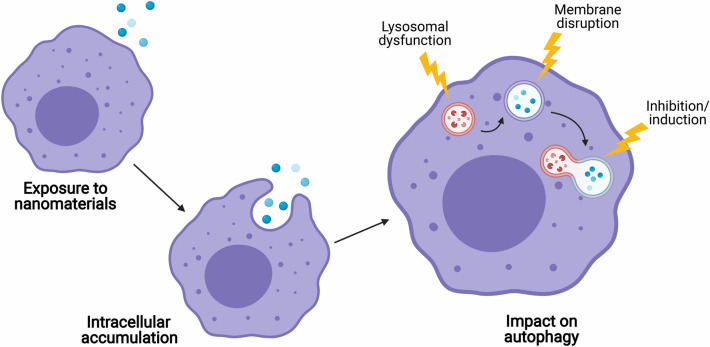

**Supplementary Information:**

The online version contains supplementary material available at 10.1186/s13578-021-00648-8.

## Background

The increase in exposure of humans to nanomaterials, through nanomedicines [1] and consumer goods [2], has emphasised the need for thorough evaluation of their potential to modulate cellular health. Biological interactions of nanomaterials are associated with a number of physicochemical properties which include core composition, coatings, hydrodynamic size, zeta potential and surface functionalisation [3]. Therefore, given the heterogeneity of engineered nanomaterials with applications in biological systems it is necessary to investigate their safety. The identification of factors linked to nanoparticle incompatibility underpins safe and rational development of nanomaterials and their subsequent translation towards commercial application.

Instances of toxicity generated by nanomaterial exposure have widely been associated to their influence on inflammation and effects on cellular processes leading to oxidative stress [4, 5]. Autophagy is intrinsically linked to these in its homeostatic role [6]. The capacity for nanomaterials to interfere with autophagic processes has been documented [7–10]. Autophagic dysfunction is becoming widely accepted as an important mechanism of nanomaterial toxicity which requires careful consideration when producing new materials [8].

Autophagy is a lysosomal degradation pathway that plays a role in maintaining homeostasis within the cell. This is accomplished through trafficking of cytoplasmic components to the autophagosome and eventual degradation for reasons including housekeeping, balancing and recycling sources of energy, and eliminating intracellular pathogens [11].

Autophagy is initiated through formation of the double-membraned autophagic vesicle that encloses ubiquitinated cellular material designated for degradation. p62/Sequestosome 1 (p62/SQSTM1) and NBR1 (Neighbor of BRCA1) interact with ubiquitinated cargoes and deliver them for autophagic degradation [12, 13]. Autophagosome formation depends on the activity of Vps34, a type III PI3K lipid kinase [14], and microtubule associated protein light-chain 3 (LC3), proteolytically activated to its membrane-bound form (LC3 II) during autophagosome assembly [15, 16]. The autophagic vesicle is subsequently fused with lysosomes for degradation.

Cell death can be caused when autophagy becomes dysregulated [17]. This has been observed in both autophagic hyperactivation [18], and autophagic failure which led to a lysosome-dependent form of cell death [19]. Dysregulation of autophagy has been linked to numerous pathological conditions including infections [20], neurodegeneration [21], Crohn’s disease [22], heart disease [16], and cancer [23, 24].

The importance of autophagy as a potential mechanism for nanoparticle toxicity has been highlighted [9]. Several nanomaterials impact autophagic processes, potentially inducing cytotoxicity; examples of which include 115 nm iron oxide nanoparticles in A549 adenocarcinomic human alveolar basal epithelial cells [25], 22 nm gold nanoparticles in MRC-5 human diploid lung fibroblast cell line [26], 54 nm polymeric nanoparticles in NR8383 rat alveolar macrophage cell line [27] and 60 nm silica in L-02 human fetal hepatocyte line and HepG2 human hepatoma cell line [7]. Numerous mechanisms for nanomaterial-mediated modulation of autophagy have been presented including defective autophagy [28], lysosomal dysfunction [7], inhibition of autophagic flux [29], and inhibition of autophagosomal degradation [7]. For this reason, it forms part of the standardised assay cascade performed by the US National Cancer Institute Nanotechnology Characterization Laboratory [30, 31]. However, the significance of nanomaterial-related autophagic dysfunction remains unclear, particularly clinically.

The aims of this work were to observe potential differences in autophagic impact by a small panel of nanomaterials varying in material, and physicochemical properties, utilising relatively facile methodologies. In addition to this, the responses to rapamycin and chloroquine by three immune cell lines: CEM (T cell), Raji (B lymphocyte), and THP-1 (human monocytic cell line) were assessed to determine the transferability of assay protocols, or impacts, between differenct cell types.

## Results

### Assessment of the impact of inducers of autophagy in cell lines over time

**Fig. 1:**
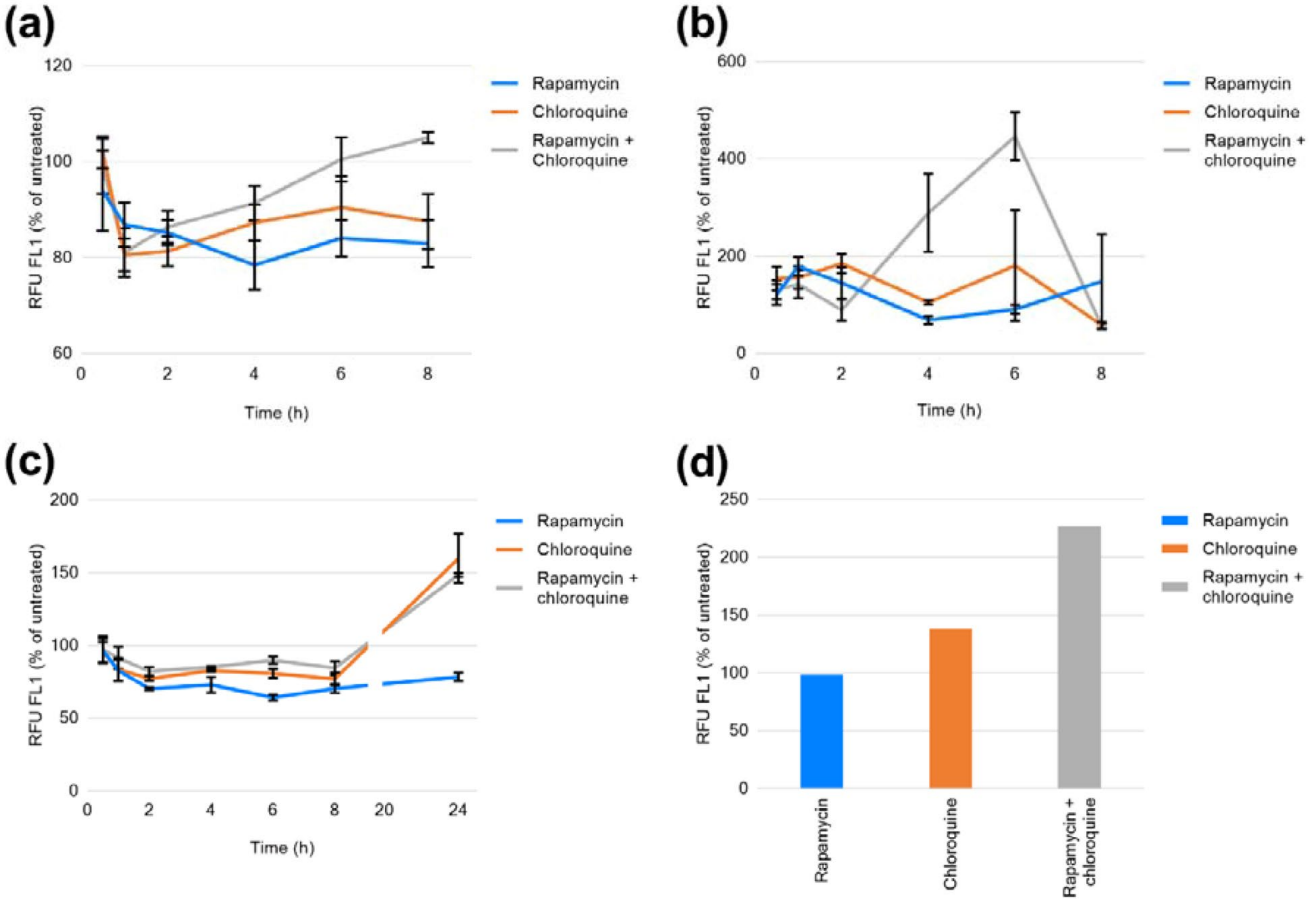
Autophagic responses, as a percentage of untreated of control, at stated time points following treatment with 0.5 µM rapamycin, 10 µM chloroquine or combined rapamycin and chloroquine in cell lines. **a** CEM. **b** Raji. **c** THP-1. Data displayed as average (n = 4) ± standard deviation. **d** Example response in Jurkat cells treated with 0.5 mM rapamycin, 10 mM chloroquine or combined rapamycin and chloroquine at 18 h [32]

Multiple time points were performed to establish optimum durations of incubation with inducers of autophagy to gain maximum observable effects, in addition to determining differences in effect between cell lines (Fig. 1a–c). The presented data demonstrate that for this particular experimental design incubation for 8 h in CEM, 6 h in Raji, and 24 h in THP-1 is necessary to provide optimal fluorescence detection.

The maximum autophagic induction in CEM and Raji (Fig. 1a, b) was produced by the combination of rapamycin and chloroquine (105 and 446 %, respectively). THP-1 demonstrated a 149 % increase under the combination, while chloroquine alone resulted in a 160 % induction (Fig. 1c). Example data provided by Enzo Life Sciences (Fig. 1d) generated in Jurkat (human T lymphocyte) shows a maximum increase in fluorescence of 117 % under exposure to rapamycin and chloroquine, validating the effectiveness of the controls observed in CEM, Raji, and THP-1.

### Impact of nanomaterials on THP-1 autophagy

**Fig. 2:**
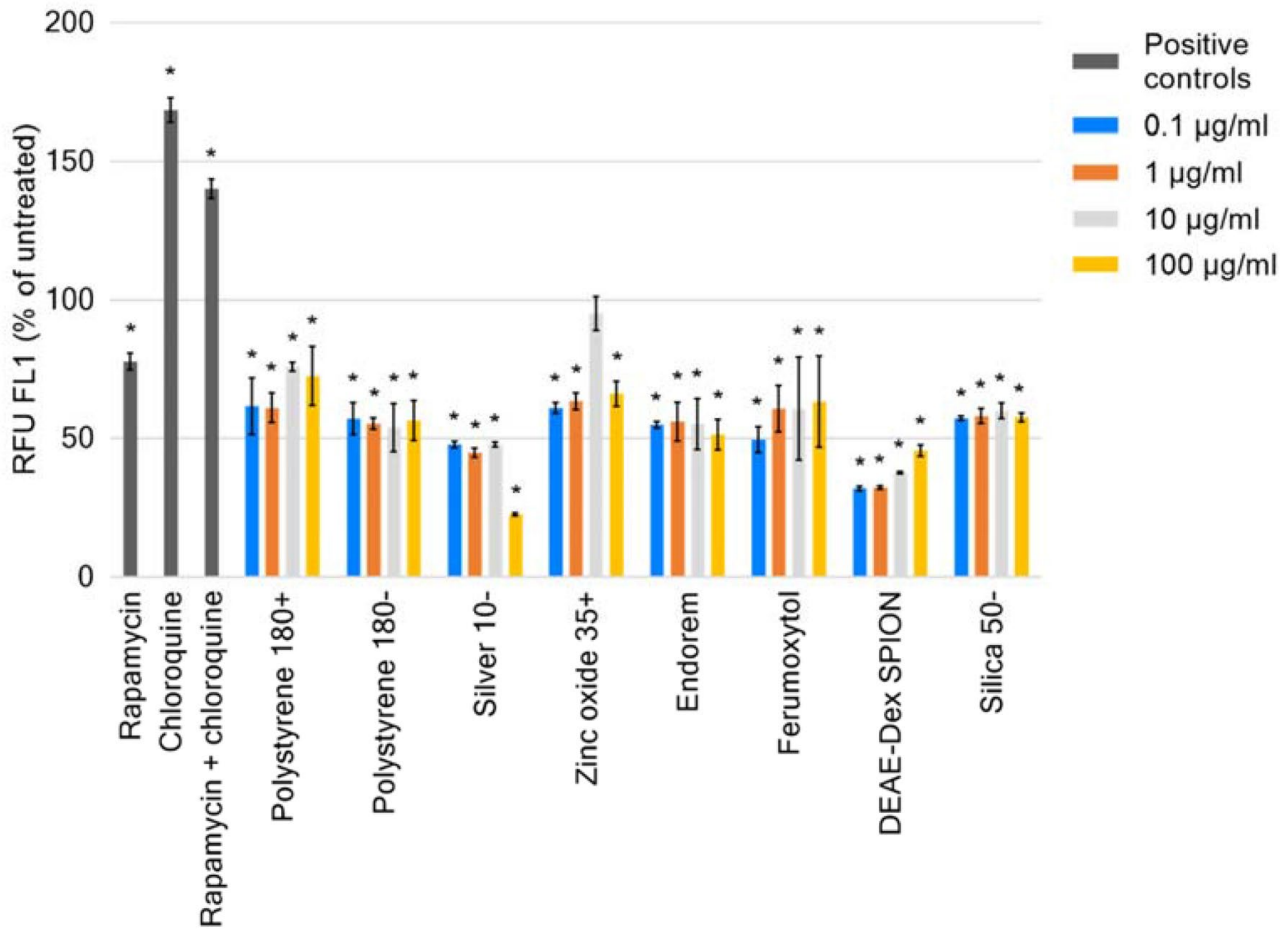
Autophagic responses of THP-1 cell line, as a percentage of untreated of control, at 24 h points following treatment with 0.5 µM rapamycin, 10 µM chloroquine, combined rapamycin and chloroquine, and nanomaterials at stated concentrations. Data displayed as an average of 4 technical replicates ± standard deviation. Asterisk denotes p < 0.05

Positive controls chloroquine, and combined rapamycin and chloroquine led to fluorescence values 40 % (p < 0.0001) higher than those of untreated THP-1. It was found that all nanomaterial treatments resulted in relative fluorescence less (p < 0.05) than that of the untreated control with the exception of 10 µg/ml zinc oxide 35 + (Fig. 2). The greatest impact was by treatment with 100 µg/ml of silver 10-, 77.3 % (p < 0.000001) less than the untreated control. An explanation, for these results, may be enhanced autophagic clearance, inhibition of the formation of the autophagosome, or disruption of autolysosomes leading to leakage of fluorescent dye from these organelles.

### Relationship between particle characteristics and autophagic impact

**Fig. 3:**
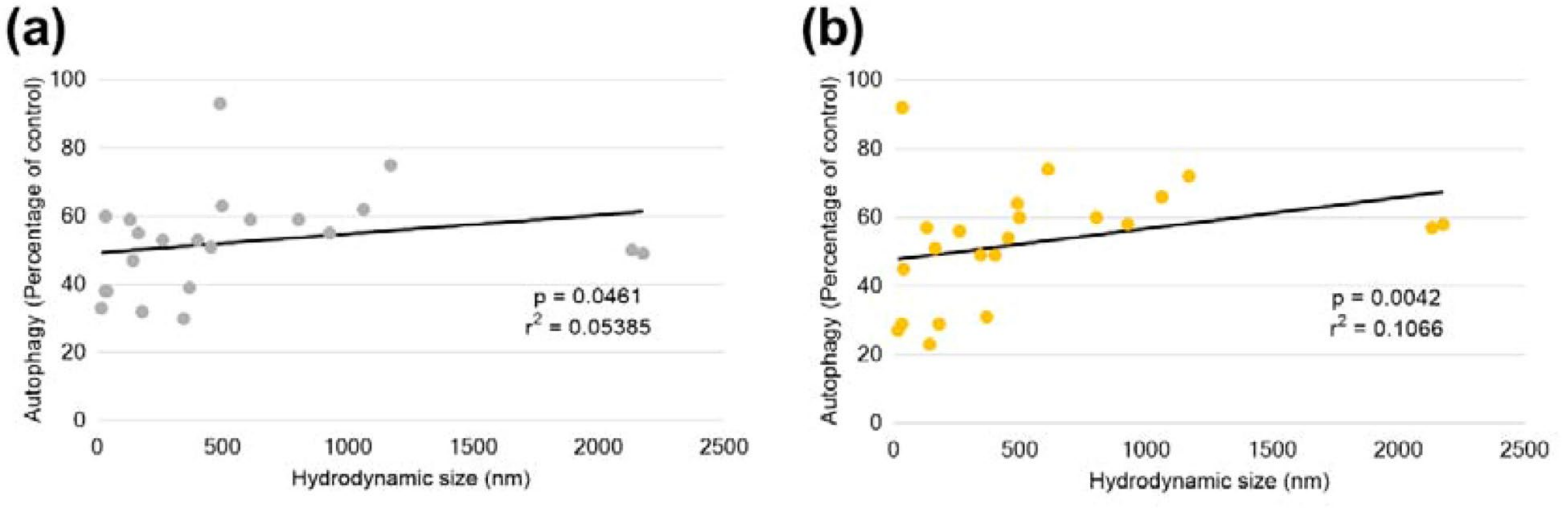
Relationship, determined via nonparametric Spearman correlation, between autophagy and nanoparticle hydrodynamic size, determined by dynamic light scattering in RPMI-1640 supplemented to 10 % final volume with FBS, at nanoparticle concentrations of (**a**) 10 µg/ml. **b** 100 µg/ml

A, statistically significant, relationship was observed between impact on autophagy and nanoparticle hydrodynamic size at nanomaterial concentrations of 10 µg/ml (p = 0.046, r^2^ = 0.054, Fig. 3a) and 100 µg/ml (p = 0.0042, r^2^ = 0.11, Fig. 3b) when assessing an extended panel of nanomaterials. This relationship demonstrate that nanoparticles of smaller size result in a greater impact on autophagy than those of larger size.

No, statistically significant, relationship was observed between nanoparticle zeta potential and impact on autophagic processes with any of the concentrations assessed here e.g. p = 0.68, r^2^ = 0.0043 at nanomaterial concentrations of 10 µg/ml.

### Evaluation of THP-1 zinc content following exposure to zinc oxide nanoparticles by inductively coupled plasma mass spectrometry (ICP-MS)

Zinc (Zn) content of RIPA buffer was found to be around 3 µg/kg; this is greater than the limit of quantification (LOQ) while being approximately 3-times less than the Zn content of the cell lysates, allowing the quantification of Zn in the cell lysates.

**Fig. 4:**
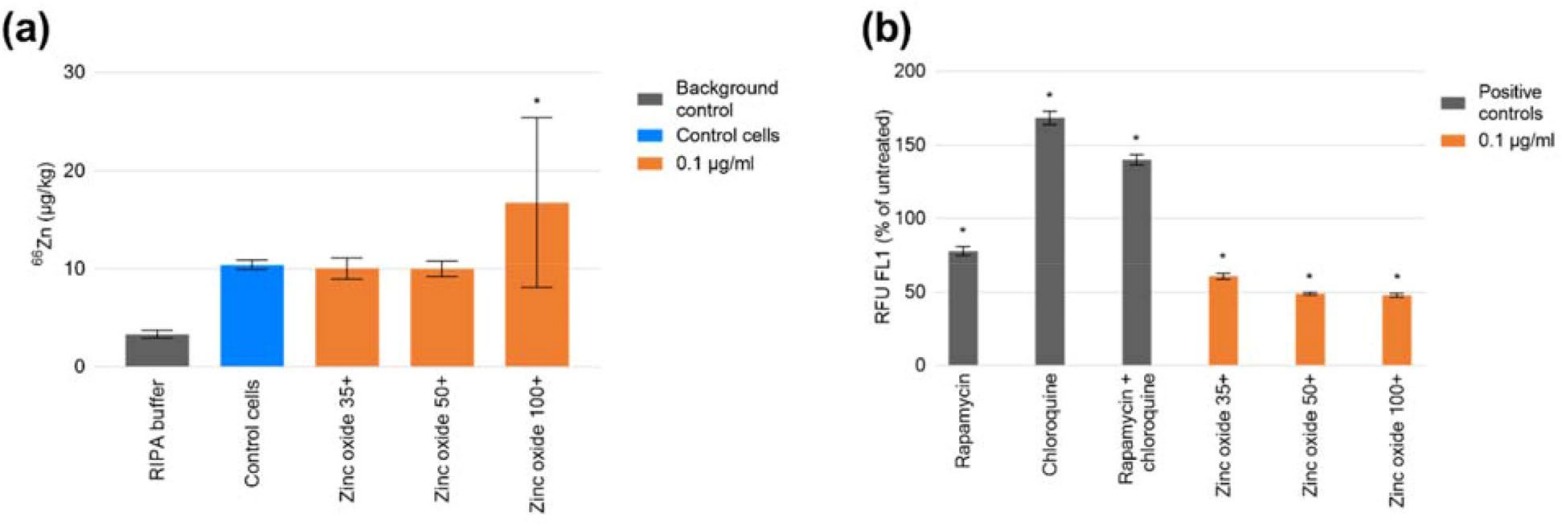
**a** Total THP-1 Zn content following exposure to 0.1 µg/ml zinc oxide nanoparticles of stated sizes determined by ICP-MS. Data displayed as average of two replicates each comprising 5 independent measurements ± standard deviation. Asterisk denotes p < 0.05. **b** Autophagic responses of THP-1 cell line, as a percentage of untreated of control, at 24 h points following treatment with 0.5 µM rapamycin, 10 µM chloroquine, combined rapamycin and chloroquine, and zinc oxide nanomaterials at 0.1 µg/ml. Data displayed as an average of 4 technical replicates ± standard deviation. Asterisk denotes p < 0.05

While exposure to the zinc oxide nanoparticles resulted in relative fluorescence 50 % less (p < 0.05) than that of the untreated control (Fig. 4b), no significant differences in Zn content were found between control cells and those exposed to zinc oxides 35 + or 50 + at a concenctraion of 0.1 µg/ml (Fig. 4a).

It should be noted that significant differences in Zn concentrations were observed for the preparation replicates of cells exposed to zinc oxide 100 +. This could, potentially, be attributed to sample inhomogeneity and or sub-sample contamination with Zn. However, the relative standard deviations (RSDs) for 5 independent measurements of each of the sub-samples are within 5 %.

Quality control samples (RIPA buffer spiked with ~ 2 and 4 µg/kg Zn) were measured in the analytical run and the recoveries, after subtraction of Zn content in the lysis buffer, averaged 80 ± 1 % and 83 ± 1 %, respectively.

### Impact of nanoparticles on p62

**Fig. 5:**
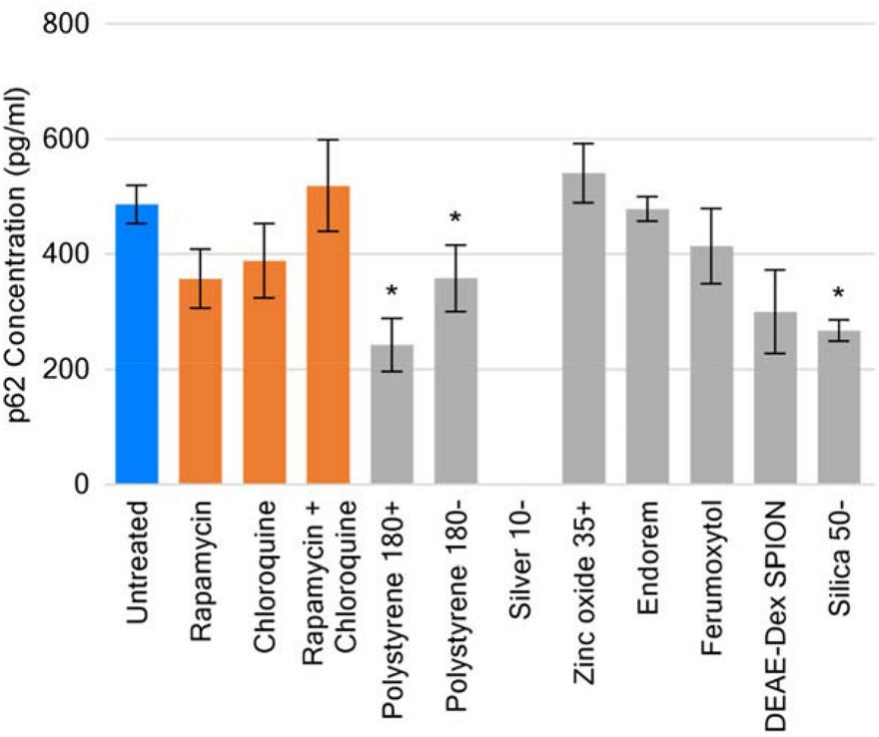
Concentration of p62 in THP-1 cells following 24-h treatment with the stated small molecules or nanomaterials. p62 concentration following exposure to silver 10- was < LOQ for the assay. Data displayed as an average of 3 technical replicates ± standard deviation. Asterisk denotes p < 0.05

The combined control of rapamycin and chloroquine, as well as zinc oxide 35 + generated levels of p62 higher than that of the untreated control, although not statistically significant. Treatment of THP-1 cells with polystyrenes 180 +, 180 −, and silica 50- led to p62 concentrations significantly lower than that of the untreated control (50 %, 74 %, and 55 %, respectively, Fig. 5), while silver 10-resulted in a p62 concentration below the LOQ for the assay.

## Discussion

The autophagic responses of three immune cell lines, CEM (T lymphocyte), Raji (B lymphocyte), and THP-1 (human monocytic cell line), were investigated at various time points to observe any potential differences in onset time and magnitude of effect, resulting from treatment with assay controls rapamycin and chloroquine.

Rapamycin, shown previously to activate autophagy in vitro and in vivo [33], was utilised to inhibit mTOR, a suppressor of autophagy [34]. mTOR, specifically the mTORC1 complex is a negative regulator of autophagy through phosphorylation of ULK1, a serine/threonine-protein kinase involved in autophagic response to starvation, under nutrient sufficiency. Additionally, mTOR inhibits autophagy through phosphorylation of DAPk (death-associated protein kinase), an important regulator of autophagy [35]. DAPk promotes autophagy through activation of Beclin 1 allowing its dissociation from the inhibitor Bcl-2 [36]. Chloroquine, an agent that impairs lysosomal acidification, has an inhibitory effect on autophagy. Through raising of lysosomal pH, fusion of lysosomes with autophagic vesicles is impaired, as well as lysosomal degradation of autophagic cargo [37].

Promotion of autophagy via rapamycin and inhibition of complete processing by chloroquine intended to generate quantifiable differences in the level of autophagy compared to untreated controls. This was achieved as displayed in Fig. 1. The flow cytometric assay chosen exploits a cationic dye which partitions into autophagic vesicles allowing quantification of relative fluorescence per treatment and comparisons between conditions. As demonstrated here, the duration of exposure required to generate the greatest observable effect differs greatly between cell types ranging from 6 to 24 h highlighting the need for similar optimisation of the assay when translating this chosen assay to other immunologically relevant cell lines. Further to this, the magnitude of autophagic induction at their determined optimal time points was clearly cell type-dependent, with the greatest response seen in the Raji cell line. This could be due to differential levels of cellular accumulation of the positive controls, or variation in the levels of autophagic proteins between cell types.

Monocytes are a phagocytic cell-type accounting for ~ 10 % of circulating white blood cells. THP1, as a model for this cell type, is prevalent in the literature for assessing interactions with nanomaterials. This is supported by their ability to demonstrate processes pertinent to the effects of nanomaterials including generation of reactive oxygen species [38], and expression of inflammasomes [39]. At the chosen time-point of 24 h, a significant modulation of autophagy in THP-1 by nanomaterials was observed with both size and concentration emerging as important nanoparticle correlates. Both the flow cytometric and ELISA methodologies exploited here would indicate a greater rate of autophagic clearance. The relative fluorescence present in cells treated with the nanomaterials would suggest fewer autophagic vesicles present in the cell available for staining. Similarly, under normal autophagic conditions p62 undergoes degradation, allowing its cellular concentration to be used as monitor of autophagic flux [34, 40]. Increased levels of this would indicate impairment or blockage of the autophagic process, as demonstrated by the combined positive control treatment. As mentioned earlier, the generated responses to nanomaterials could indeed be the result of enhanced autophagic flux, but it is possible that the nanomaterials cause disruption to autolysosomes leading to leakage of fluorescent dye from these organelles, or interfere with autophagosomal formation via key proteins in the process. These alternative mechanisms have been described for nanomaterials such as silica [7], and while possible are not conclusively occurring under the tested conditions. Therefore, further investigation is required.

It is well-known that nanomaterial size is a key determinant for effective cellular uptake and accumulation [41]. Zinc oxide, chosen as an exemplar in this study, did not demonstrate any significant accumulation when compared to the Zn content of untreated cells at sizes of 35 and 50 nm. This serves to highlight that the observed modulation of autophagy (Fig. 4b) is multifactorial in nature, potentially involving subcellular localisation of nanoparticles, interference with signal transduction, ion release, etc.

The hypothesis of enhanced autophagic induction is supported in the literature with the example of the nanomaterial which generated the greatest observed effect; silver nanoparticles [42]. Combined exposure with nanomaterials and inducers/inhibitors of autophagy could potentially enhance or unmask effects that are unobservable under the conditions described in this study. However, such an investigation was beyond the scope of current work that served as a screen for autophagic impact.

As mentioned earlier, the potential for nanomaterials to affect cellular health through their impact on pro- and antioxidant mechanisms is well known [43]. Oxidative stress is regulated through autophagy so as to maintain homeostasis [17]. In addition to the direct implications of reactive oxygen species on intracellular organelles, they have been linked to the upregulation of proinflammatory cytokines [44]. Autophagy is a known mechanism through which inflammatory markers are regulated [45], and plays a role in apoptosis, necrosis, necroptosis, and pyroptosis [46]. For disease states in which autophagy has become dysregulated, modulation of autophagic mechanisms may present an effective treatment target. This strategy is being employed in the design of novel chemotherapeutics [47, 48]. Nanomaterials capable of influencing autophagic flux would be of great utility in these efforts providing multipotent platforms for biomedical applications and therapeutics. However, for the most efficacious application of nanomaterials as autophagic modulators, the intrinsic status of these processes must be considered, and the nanomaterial impact should be balanced against its impact on cellular health and inflammation.

## Conclusions

The utility of cell lines and assays which serve as a platform to observe nanomaterial influence on autophagy is demonstrated here. The inclusion of additional nanomaterials, with robust physico-chemical characterisation, would serve to improve the analysis of relationships between characteristics and effect. It is clear that these nanomaterials impact on autophagy, within the cells studies. Further investigation into the mechanisms behind this, and if the results translate to primary cell counterparts, is now underway. However, the data presented here show that these models have utility in the “safe-by-design” of nanomaterials, particulary in reference to their impact on autophagic processes.

## Methods

### Materials

The source and manufacturer stated physical characteristics of the nanoparticles, used in the current study are summarised in Table 1. RPMI-1640, phosphate buffered saline (PBS), foetal bovine serum (FBS), chloroquine, and rapamycin were purchased from Sigma-Aldrich (Dorset, UK). CYTO-ID Autophagy Detection Kit and p62 ELISA Kit were purchased from Enzo Life Sciences (Exeter, UK). MACSQuant running buffer was purchased from Miltenyi Biotec GmbH (Bergisch Gladbach, Germany). RIPA lysis and extraction buffer was purchased from Thermo Fisher Scientific (Altrincham, UK). CEM, Raji, and THP-1 cell lines were purchased from ECACC (European collection of cell cultures) via Public Health England (Salisbury, UK).

**Table 1:** Summary of the physical characteristics of the nanoparticles used in the present study

Designation	Size (nm)	Zeta potential (mV)	Stabiliser	Surface groups	Source
Polystyrene 180+	180	52.2	N/A	Quaternary ammonium	Sciventions
Polystyrene 180−	180	− 40.9	N/A	Sulphonate	Sciventions
Polystyrene 275+	275	42.2	N/A	Quaternary ammonium	Sciventions
Polystyrene 300–	300	− 37.2	N/A	Sulphonate	Sciventions
Polystyrene 440+	440	39.2	N/A	Quaternary ammonium	Sciventions
Polystyrene 440−	440	− 34.9	N/A	Sulphonate	Sciventions
Gold 10−	10	N/A	Sodium citrate	N/A	BBInternational
Gold 11 –	11	N/A	N/A	HS-PEG and peptidols	University of Liverpool [49]
Silver 10−	10	− 39.0	Sodium polyacrylate	N/A	Sciventions
Silver 20+	20	N/A	Sodium citrate	N/A	Sigma-Aldrich
Endorem	5	– 6.8	Sodium citrate	Mannitol	Guerbet GmbH
Ferumoxytol	30	− 30.6	N/A	Carboxyl	AMAG Pharmaceuticals
DEAE-Dex SPION	6.9	20.2	N/A	Diethylaminoethyl dextran	University of Liverpool [50]
Titanium (IV) oxide 21+	21	N/A	N/A	N/A	Sigma-Aldrich
Zinc oxide 35+	35	N/A	3-Aminopropyl triethoxysilane	N/A	Sigma-Aldrich
Zinc oxide 50+	50	N/A	N/A	N/A	Sigma-Aldrich
Zinc oxide 100+	100	N/A	N/A	N/A	Sigma-Aldrich
Silica 50-	50	− 34.7	l-arginine	N/A	Sciventions
Silica 310–	310	− 37.1	l-arginine	N/A	Sciventions
Silica 100+	100	N/A	N/A	N/A	Invivogen

### Routine cell culture

CEM, Raji, and THP-1 cells were maintained in RPMI-1640 media supplemented with 10 % v/v FBS and incubated at 37 °C, 5 % CO_2_. All assessments were performed at passage < 15.

### Assessment of the impact of inducers of autophagy in cell lines over time

CEM, Raji, and THP-1 cells (5 × 10^5^ per well) were incubated with rapamycin (500 nM), chloroquine (10 µM), or both in 96-well plates. Time points chosen were 0.5, 1, 2, 4, 6, and 8 h with an additional 24-h exposure time in the THP-1 cell line. Following incubation, autophagy was measured using CYTO-ID autophagy detection kit as per manufacturer’s instructions for flow cytometric analysis. Quantification was performed via flow cytometry (MACSQuant, Miltenyi Biotec, Germany). The gating strategy employed here isolated the viable cell population, based on forward and side scatter characteristics and mean fluorescence was recorded from the green (FL1) channel.

### Impact of nanomaterials on THP-1 autophagy

Nanoparticles of various composition, size, charge, and functionalization were assessed on their impact on the autophagic process in THP-1 cell line. Nanomaterials were assayed in an identical manner as the autophagic inducers described above at concentrations of 0.1, 1, 10, and 100 µg/ml with an exposure time of 24 h.

### Evaluation of THP-1 zinc content following exposure to zinc oxide nanoparticles by inductively coupled plasma mass spectrometry (ICP-MS)

THP-1 cells were seeded at 5 × 10^5^ cells/ml to 12-well plates. Cells were treated with 0.1 µg/ml zinc oxide 35+, 50+, and 100 + nanoparticles for a period of 24 h. Untreated control cells were prepared in parallel. Cells were collected, washed three times with PBS, and subsequently lysed using 0.5 ml of RIPA buffer and stored at − 80 °C.

Samples were diluted 1:20 with ultrapure water (18 MΩ cm, Veolia Water Technologies, High Wycombe, U.K.) containing germanium (Ge) as an internal standard. Two independent replicates were prepared for each sample. For quality purposes and to assess method accuracy, two solutions containing known amounts of inorganic Zn (~ 2 and 4 µg/kg Zn) were prepared in RIPA buffer to mimic lysis conditions.

Quantification was performed via external calibration using commercial Zn and Ge calibration standards from an accredited supplier (Romil, UK). Zn calibration standards were prepared matching sample conditions in ultrapure water containing Ge as an internal standard, to correct for any instrumental drift or ionisation effects. The external calibration ranged from 0.1 to 10 µg/kg of Zn and ~ 9 µg/kg of Ge. Calibration curves achieved correlation coefficients of at least 0.995.

Samples analysis was performed using a collision/reaction cell ICP-MS 8800 ICP-MS/MS (Agilent Technologies, UK) in helium (He) gas mode. The samples were introduced into the plasma via a MicroMist quartz concentric nebuliser, operating in pumping mode at 0.1 rps, and a Scott double pass spray chamber cooled to 2 °C. The instrument was tuned prior to analysis for optimum signal intensity and stability, with typical operating parameters provided in Additional file 1: Table S1. During analysis, up to six samples were bracketed by 2 blank measurements and a “check” standard, typically the middle calibration standard, to ensure there was no drift.

The LOD/LOQ was evaluated using at least 6 measurements of the reagent blank in the analytical run. The LOD and LOQ were calculated as the mean blank concentration plus 3 and 10 times respectively, the standard deviation of the blank measurements. All sample results were corrected for individual dilution factors. Total Zn mass fractions (µg/kg) are reported for isotope ^66^Zn (27.9 % abundance). Although this isotope is less abundant than ^64^Zn (48.6 %), detection of the latter is likely to be affected by spectral interferences, especially ^16^Ar^14^N_2_+ and ^64^Ni. Results obtained for the other isotope of Zn (^67^Zn, 4.1 %) were in good agreement with those reported. The reported LOD and LOQ for ^66^Zn were based on an average 19-fold dilution of the samples.

### Impact of nanoparticles on p62

THP-1 cells were seeded to 12-well plates at a density of 5 × 10^5^ cells/ml. Cells were treated with rapamycin (0.5 µM), chloroquine (10 µM), rapamycin and chloroquine, and a selection of nanomaterials (100 µg/ml) for a period of 24 h. The concentration of p62 was measured in cell lysates using the p62 ELISA Kit following the manufacturer’s protocol.

### Statistics

Graphs and statistical analyses were performed with GraphPad Prism 7 and/or Microsoft Excel 2016. All data is displayed as an average ± standard deviation. Differences between controls and treatments were evaluated by t test or linear regression. Statistical significance was considered at P < 0.05.

## Supplementary Information

**Additional file 1: Table S1. **General ICP-MS parametersused for Zn analysis.

## Data Availability

The datasets used and/or analysed during the current study are available from the corresponding author on reasonable request.

## Abbreviations

Bcl-2: B-cell lymphoma 2
DAPk: Death-associated protein kinase
ELISA: Enzyme-linked immunosorbent assay
FBS: Foetal bovine serum
Ge: Germanium
He: Helium
ICP-MS: Inductively coupled plasma mass spectrometry
LC3: Microtubule-associated protein 1 A/1B-light chain 3
LOD: Limit of detection
LOQ: Limit of quantification.
mTOR: Mammalian target of rapamycin
NBR1: Neighbour of BRCA1
p62/SQSTM1: Sequestrome 1
PEG: Polyethylene glyco
PBS: Phosphate buffered saline
RIPA: Radioimmunoprecipitation assay
RPS: Relative pressure sensor
RSDs: Relative standard deviations
RPMI: Roswell Park Memorial Institute
Vps34: Vacuolar protein sorting 34
Zn: Zinc
