# Supplementary material for: Assessment of changes in autophagic vesicles in human immune cell lines exposed to nano particles
Source: Cell Biosci. 2021 Jul 16;11:133. doi: 10.1186/s13578-021-00648-8 (PMC8283997; doi:10.1186/s13578-021-00648-8)
Supplement: Supplementary file 1 — Additional file 1: Table S1. General ICP-MS parametersused for Zn analysis. [file 13578_2021_648_MOESM1_ESM.docx]

Table S1: General ICP-MS parameters used for Zn analysis

| **Agilent 8800 ICP-MS/MS** | |
| --- | --- |
| *General Conditions:* | |
| RF Power | 1550 W |
| Plasma Gas | 15 L/min |
| Auxiliary Gas Flow | 0.9 L/min |
| Carrier Gas | 1.14 L/min |
| Nebulizer Pump | 0.10 rps |
| Sample Depth | 7.0 mm |
| Spray Chamber Temperature | 2°C |
| Cell Gas Flow/Settings | 5 mL/min He |
| Measurement Mode | Single quad |
| *Measurement Conditions:* Spectrum mode | |
| Points per peak | 3 |
| Replicates | 5 |
| Sweeps/Replicate | 100 |
| Measured Isotopes | ^64^Zn, ^66^Zn, ^67^Zn, ^68^Zn, ^73^Ge |
| Dwell Time | 0.09 s all isotopes |
